# The complete chloroplast genome of *Pilea notata* C. H. Wright, 1899 (Urticaceae)

**DOI:** 10.1080/23802359.2024.2392762

**Published:** 2024-09-16

**Authors:** Ni Zhao, Linya Liu, Shudong Zhang, Chao Zhao, Xiaojian Gong, Yacheng Huang

**Affiliations:** a Key Laboratory for Information System of Mountainous Areas and Protection of Ecological Environment, Guizhou Normal University, Guiyang, China; b School of Biological Sciences and Technology, Liupanshui Normal University, Liupanshui, China

**Keywords:** Chloroplast genome, phylogenetic analysis, *Pilea notata*, Urticaceae

## Abstract

*Pilea notata* (*Pilea notata* C. H. Wright_C. H. Wright, 1899) is *Pilea Lindl*. of Urticaceae, which is a commonly used Miao medicine in Guizhou province. The *P. notata* chloroplast genome is 150,979 bp, contains a pair of inverted repeats (IRs 25,743bp), and is separated by a large single-copy region (81,446bp) and a small single-copy region (18,047bp). A total of 131 genes, including 86 protein-coding genes, 37 tRNA genes, and eight rRNA genes. Phylogenetic analysis showed that *P. notata*, *P. verrucosa* and *P. monilifera* united as a single branch, while *Pilea cadierei* was defined as a sister group of this branch.

## Graphical Abstract

**Figure:**
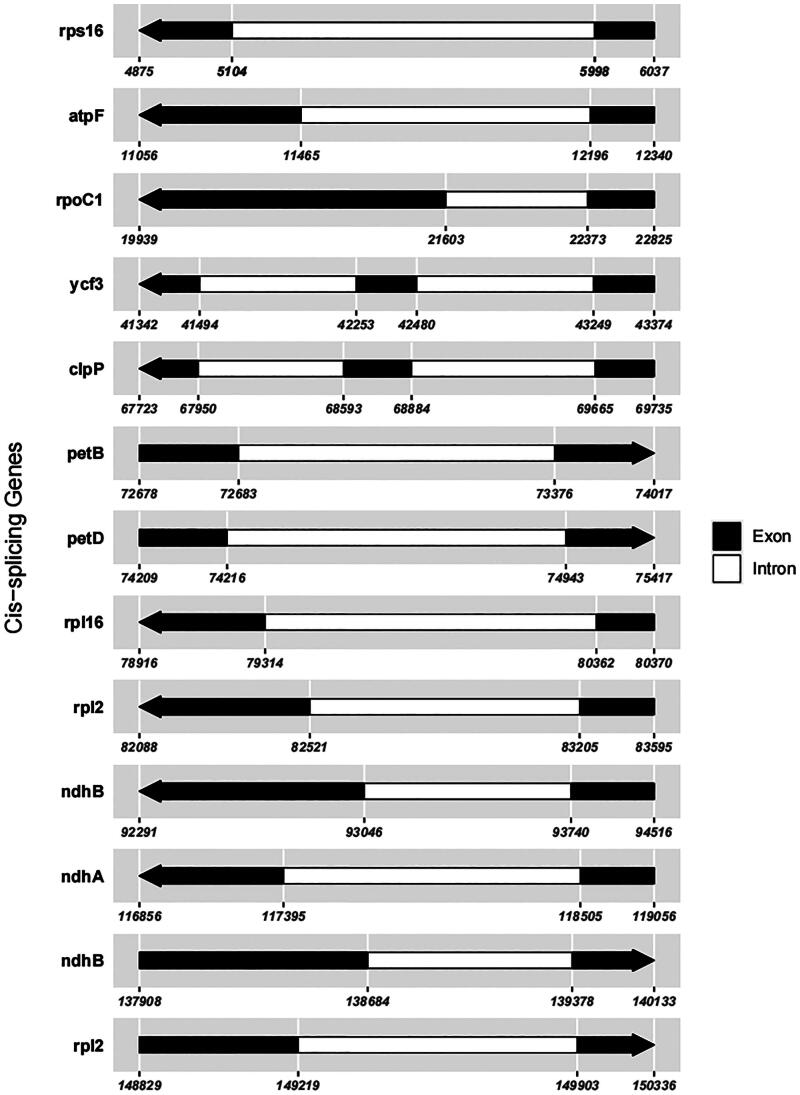

## Introduction

*Pilea notata* (*Pilea notata* C. H. Wright_ C. H. Wright, 1899) is a perennial juice-rich herb in the *Pilea Lindl* of Urticaceae (Wang and Li 2005; Ye et al. 2021), it is a dioecious plant, alias water hemp leaf, goat grass, soil licorice, white goat, sweet grass, etc. It is a well-known herbal medicine for the Hmong in Guizhou, and is called ‘SHUI WU DA’ in Guizhou Miao language (Wang and Li 2005), and functions on treating many diseases, such as hot and humid jaundice, red and white belt, shower turbidity, urine blood, children summer heat, malaria mother, indigestion, fall, injury, trauma infection and so on (Ye et al. 2021). Moreover, the extract obtained from *P. notata* also has antibacterial (Gan, Liang, and Jiang 2014; Gan, Liang, Zhao, et al. 2014; Gan et al. 2015), antioxidant (Gan et al. 2015), anti-inflammatory and analgesic (Sun et al. 2009; Guo 2018) effects. *P. notata* grows in the forest or ditch side Shady wetlands and its shade resistance ability is relatively strong and due to the deep green color of its leaves, it was rated as excellent color leaf plant resources, and could be used as indoor ornamental plants and urban greening plants (Huang 2007; Xiao 2021). In addition, it also shows good comprehensive purification ability and resistance to formaldehyde with strong carbon monoxide, carbon dioxide, and adsorption of benzene and phenol. Recent studies also suggest that *P. notata* may be a heavy metal cobalt-rich plant with some evolutionary effects on heavy metal pollution (Zhang 2011; Zhu 2011; Chen et al. 2018; Zhang et al. 2023). The complete chloroplast genome identification was supposed to reveal the systematic relationship between species more comprehensively, and the sequencing, assembly annotation, and phylogenetic analysis would facilitate the study of genetic breeding and cultivation of *P. notata*. To provide a better understanding and utilization of *P. notata*, the complete chloroplast genome of this species was firstly analyzed from high-throughput Illumina sequencing reads.

## Materials and methods

### Plant material, DNA extraction, and sequencing

**Figure 1.**
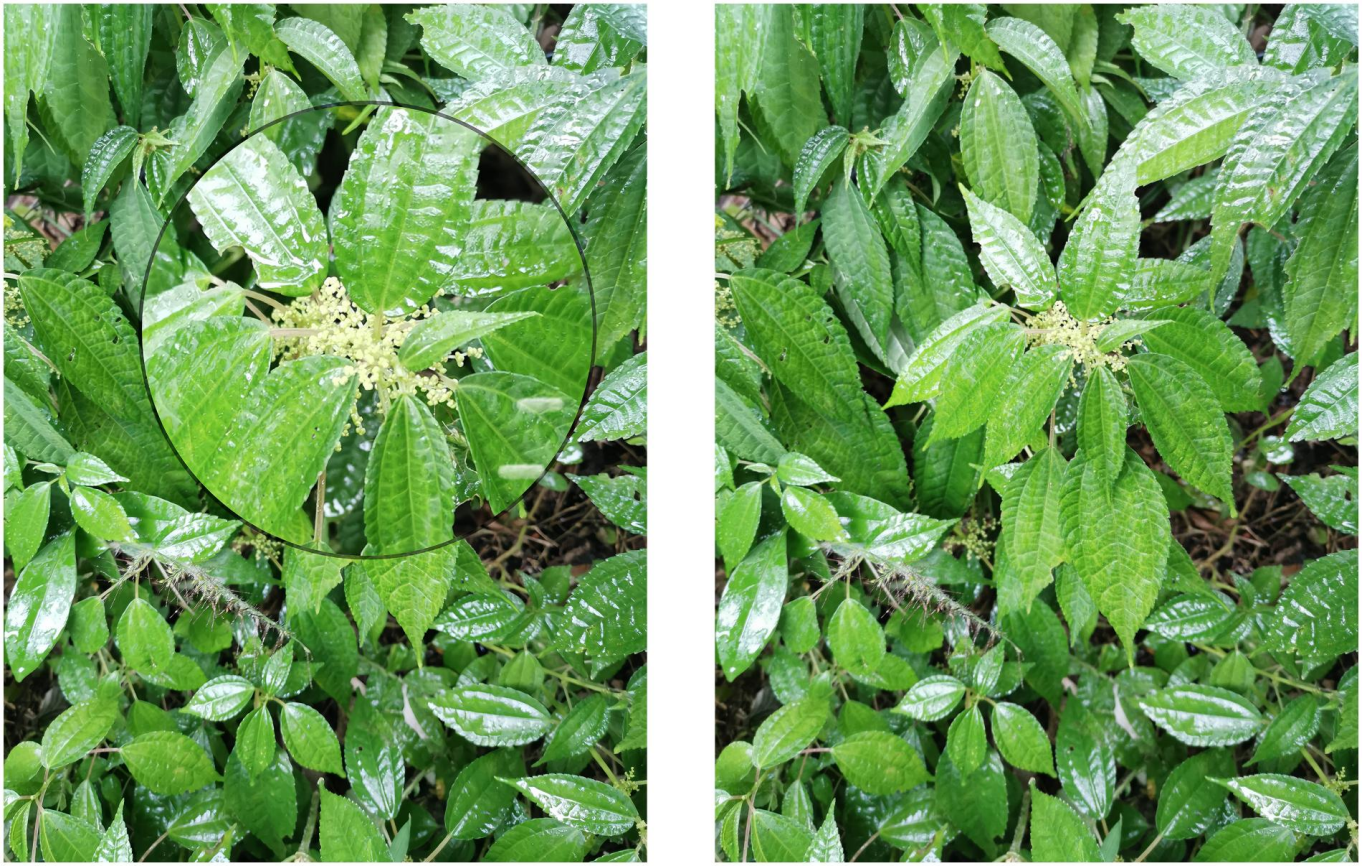
*Pilea notata* used in this study. The image was taken by Ni Zhao at Qianling Mountain, Yunyan District, Guiyang City, Guizhou Province (N26°36′5.01″, E106°41′19.90″). core features: have a creeping stem. Stem fleshy, slender, slightly swollen middle, 25–70 cm high, 2–4 mm thick, glabrous, thin upper part pubescent, densely striped stalactite. Leaves papery, nearly equal in size to the same pair, narrowly ovate, ovate-lanceolate or ovate, 4–11 cm long, 1.5–4.5 cm wide, apex caudate or acuminate, base rounded, thinly broadly cuneate, margin shallowly serrate from lower to apex, rare double serrate, dark green, glossy above, light green below, stalactite striate, 0.5–0.6 mm long, densely covered on both sides, obviously, there are three basal veins, and the two side arcs extend to the upper part and the side veins ring junction, the side veins 8–13 pairs, slightly oblique spread as network veins; petiole slender, 17 cm long, often glabrous, rarely pubescent; stipule large, greenish, oblong, 8–12 mm long, shedding. Flowers dioecious; male flower sequence corymbose raceme, 2–5 cm long, with few branches, corymbose clusters sparsely on the flower branches; the female cyme is short and dense.

The *P. notata* used in this study were collected from the Qianling Mountain, Yunyan District, Guiyang City, Guizhou Province (Figure 1; N26°36′5.01″, E106°41′19.90″, 1100 m) and a specimen was deposited at School of Biological Sciences and Technology, Liupanshui Normal University (collected by Yacheng Huang and yachenghuang1314@126.com.) under the voucher number (SKY2208). The genomic DNA was extracted and sequenced on the Illumina Novoseq 4000 platform as previously described (Zhang et al. 2019). The clean image data files obtained from high throughput sequencing were converted into 1.9 GB of clean reads by base recognition analysis, and the results were stored in FASTQ file format.

### Chloroplast genome assembly and annotation

The complete chloroplast genome of *P. notata* was *de novo* assembled using the GetOrganelle pipeline (https://github.com/Kinggerm/GetOrganelle) and all genes were annotated using CPGAVAS (Liu et al. 2012). The online tRNAscan-SE Search Service (Lowe and Chan 2016) (http://lowelab.ucsc.edu/tRNAscan-SE/) was used to further confirm tRNA genes. The circular genome map and detailed structure of the chloroplast genome were drawn using the CPGview package (Liu et al. 2023). In addition, the CPGView (Liu et al. 2023) was applied to visualize the intron-containing genes. The SAM file is generated using BWA aligner and converted into a BAM file *via* SAMtools for evaluating the depth coverage map (Li and Durbin 2009).

### Phylogenetic analysis

In this study, the 19 chloroplast genome sequence representatives of the Urticaceae were downloaded, three species of the genus Urticaceae were used as outgroup. The sequence alignment of these 19 chloroplast complete genome sequences was performed using the MAFFT online site (https://mafft.cbrc.jp/alignment/server/) (Katoh and Standley 2013) and analyzed using MEGA 7 (Kumar et al. 2016) to generate a phylogenetic tree.

## Results

### General features of the chloroplast genome

**Figure 2.**
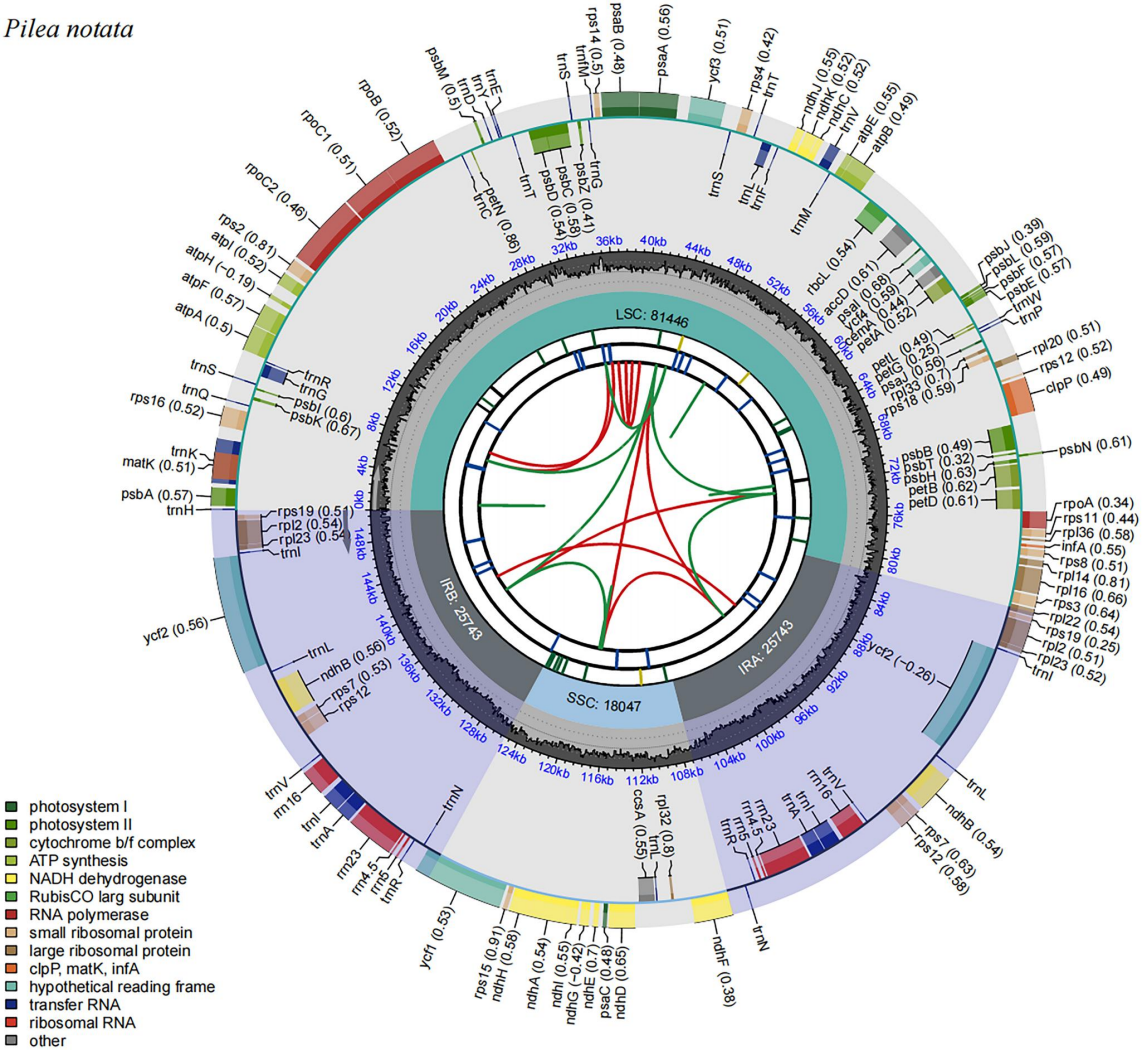
Circular map of the complete chloroplast genome of *Pilea notata* generated by CPGview. The map contains six tracks in default. From the center outward, the first track shows the dispersed repeats. The dispersed repeats consist of direct (D) and palindromic (P) repeats, connected with red and green arcs. The second track shows the long tandem repeats as short blue bars. The third track shows the short tandem repeats or microsatellite sequences as short bars with different colors. The small single-copy (SSC), inverted repeat (IRa and IRb), and large single-copy (LSC) regions are shown on the fourth track. The GC content along the genome is plotted on the fifth track. The genes are shown on the sixth track. The optional codon usage bias is displayed in the parenthesis after the gene name. Genes are color-coded by their functional classification. The transcription directions for the inner and outer genes are clockwise and anticlockwise, respectively. The functional classification of the genes is shown in the bottom left corner.

The complete chloroplast genome sequence of *P. notata* had been submitted to the GenBank database (accession number: OQ198624.1). The raw reads were deposited in the GenBank Sequence Read Archive (accession no. SRR28371196). The *P. notata* chloroplast genome was a circular genome with a full-length sequence of 150,979 bp (Figure 2) and a GC content of 37%, consisting of an 81,446 bp large single-copy region (GC content 34%) and a small single-copy region of 18,047 bp (GC content 30%), separated by a pair of identical 25,743 bp inverted repeats (GC content 43%). The complete chloroplast genome included 131 genes, 86 protein-coding genes, 37 tRNA, and eight rRNA. Most of the genes occurred in a single copy, while four rRNA genes (i.e. *4.5S*, *5S*, *16S*, and *23S* rRNA), seven tRNA genes (i.e. *trn*I-CAU, *trn*L-CAA, *trn*V-GAC, *trn*I-GAU, *trn*A-UGC, *trn*R-ACG, and *trn*N-GUU), and six protein-coding genes (i.e. *ndh*B, *rpl*2, *rpl*23, *rps*7, *rps*12, and *ycf*2) occurred in double. Nine protein-coding genes (*rps*16, *atp*F, *rpo*C1, *pet*B, *pet*D, *rpl*16, *rpl*2, *ndh*B, *ndh*A) were single-intron genes, and two protein-coding genes (*ycf*3, *clp*P) had two introns (Supplementary Figure S1 and Figure S2). And the average depth of coverage of individual bases was 227 folds (Supplementary Figure S3).

### Phylogenetic analysis

**Figure 3.**
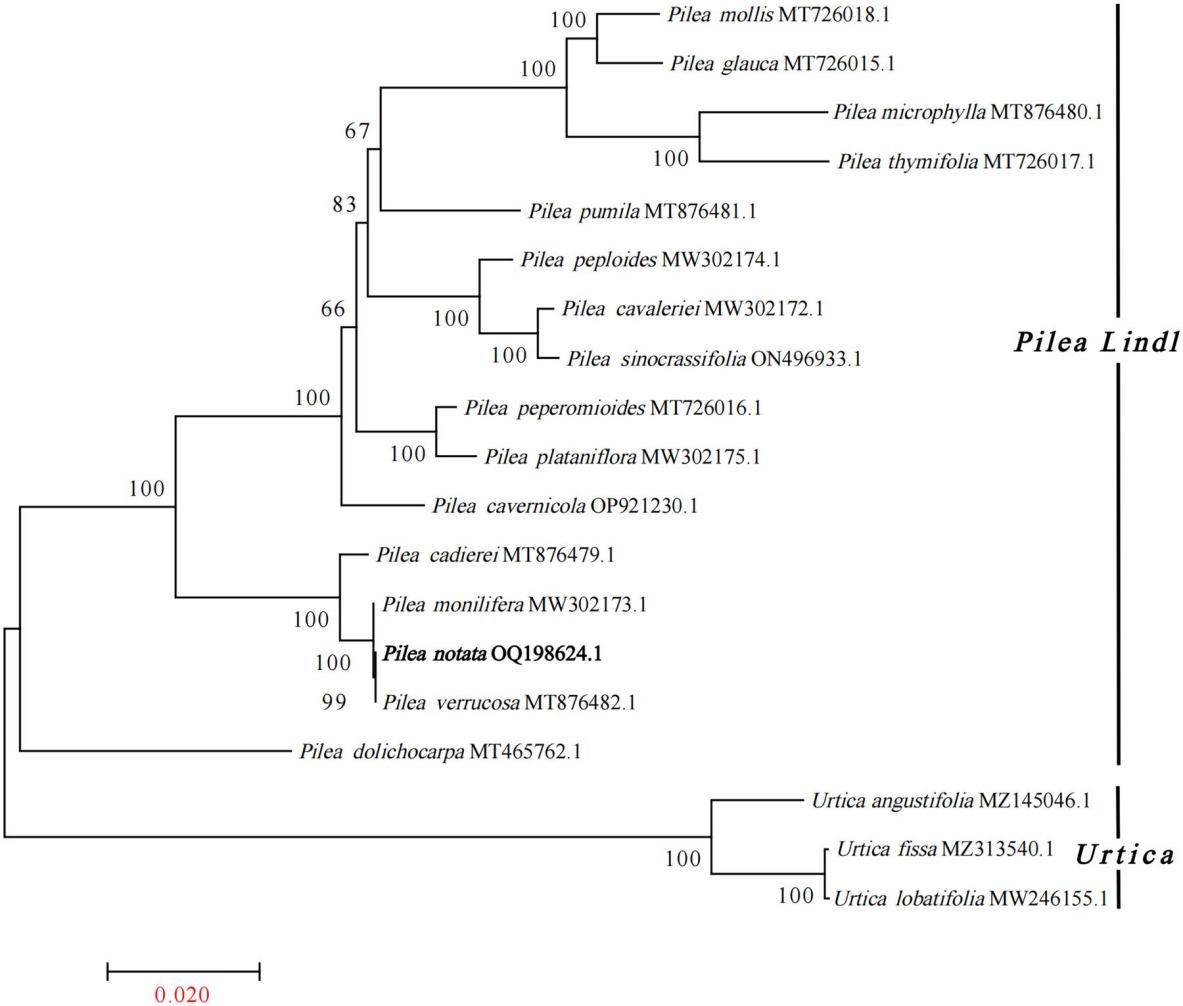
The maximum-likelihood (ML) tree of Urticaceae is inferred from the complete chloroplast genome sequences. Phylogenetic tree constructed by maximum-likelihood (ML) analysis based on complete chloroplast genome sequences, including *Pilea notata* C. H. Wright (OQ198624.1) sequenced in this study. Numbers at nodes correspond to ML bootstrap percentages (1000 replicates). The sequences used for tree construction are as follows: *Pilea notata* C. H. Wright, 1899 (OQ198624.1; this study), *Pilea mollis* (MT726018.1; Li et al. 2021), *Pilea glauca* (MT726015.1; Li et al. 2021), *Pilea microphylla* (MT876480.1), *Pilea thymifolia* (MT726017.1), *Pilea pumila* (MT876481.1), *Pilea peploides* (MW302174.1), *Pilea cavaleriei* (MW302172.1), *Pilea sinocrassifolia* (ON496933.1), *Pilea peperomioides* (MT726016.1; Li et al. 2021), *Pilea plataniflora* (MW302175.1), *Pilea cavernicola* (OP921230.1), *Pilea cadierei* (MT876479.1), *Pilea monilifera* (MW302173.1), *Pilea verrucosa* (MT876482.1), *Pilea dolichocarpa* (MT465762.1), *Urtica angustifolia* (MZ145046.1; Liu et al. 2023), *Urtica fissa* (MZ313540.1; Li et al. 2022), and *Urtica lobatifolia* (MW246155.1).

*Pilea notata* is a perennial herb of the *Pilea Lindl* of Urticaceae. The sequence alignment of these 19 chloroplast complete genome sequences was performed using the MAFFT online site (https://mafft.cbrc.jp/alignment/server/) (Katoh and Standley 2013) and analyzed using MEGA 7 (Kumar et al. 2016) to generate a phylogenetic tree. The phylogenetic analysis showed high bootstrap values for most of the nodes in the phylogenetic tree (Figure 3). Of their phylogenetic position, as shown in the phylogenetic tree, *P. notata, Pilea verrucosa,* and *Pilea monilifera* united as a single branch, and *P. notata* was mostly related to *P. verrucosa*, with a bootstrap support value of 99%, while *Pilea cadierei* was defined as a sister group of this branch. This study adds to our understanding of the origin and evolution of *P. notata*, as well as its genetic links with other species.

## Discussion

Compared with nuclear and mitochondrial genomes, chloroplast genomes are highly conserved and widely used in phylogenetic and evolutionary studies. There are over 447 species in this genus, distributed in tropical and subtropical regions of the world, and about 90 species in China (Chen et al. 2018). Phylogenetic analysis was performed to understand the phylogenetic relationship of *P. notata* with other species. With the development of high-throughput sequencing technology, the chloroplast genome sequence plays an important role in species identification as a super barcode (Li et al. 2021). In this study, phylogenetic relationship based on the complete chloroplast sequences showed high bootstrap support. The phylogenetic tree was in agreement with the previous studies, the results of Li and Yang et al. showed that *P. monilifera*, *P. verrucosa* united as a single branch, while *P. cadierei* was defined as a sister group of this branch, which was consistent with the results of this study (Li et al. 2022; Yang et al. 2022). So this study contributed vital data for *Pilea Lindl* genus taxonomy. Significantly, it enhanced the understanding of the phylogenetic relationships within the Urticaceae, and facilitated to develop species-specific molecular markers, which was crucial for Urticaceae.

## Conclusion

This study is the first report of the complete chloroplast genome sequence of *P. notata*. In the phylogenetic tree, *P. notata, P. verrucosa,* and *P. monilifera* united as a single branch, while *P. cadierei* was defined as a sister group of this branch. The *P. notata* cp genome reported in this study provided a useful resource for future in-depth research on the ornamental and ecological value of the development and phylogeny of *P. notata.* In addition, the results would facilitate the development of species-specific markers to authenticate *P. notata* herbal drug at the variety level.

## Ethical approval

No permission was required to collect *P. notata* because it is widely distributed in the forest or ditch side Shady wetlands. The plant species was collected from the Qianling Mountain, Yunyan District, Guiyang City, Guizhou Province (GPS coordinates: N26°36′5.01″, E106°41′19.90″).

## Supplementary Material

GraphicalAbstractS2.png

GraphicalAbstractS1.png

GraphicalAbstractS3.png

## Data Availability

The data that support the findings of this study are openly available in NCBI (https://www.ncbi.nlm.nih.gov/). The complete chloroplast genome of *P. notata* was deposited in GenBank under the accession OQ198624.1 (https://www.ncbi.nlm.nih.gov/nuccore/OQ198624.1). The associated high throughput sequencing data files are available from the BioProject, Bio-Sample, and SRA submission under the accession numbers PRJNA1087281, SAMN40436524, and SRR28371196, respectively.
