# Supplementary figures and images for: The complete chloroplast genome of Pilea notata C. H. Wright, 1899 (Urticaceae)
Source: Mitochondrial DNA B Resour. 2024 Sep 16;9(9):1237–42. doi: 10.1080/23802359.2024.2392762 (PMC11409408; doi:10.1080/23802359.2024.2392762)

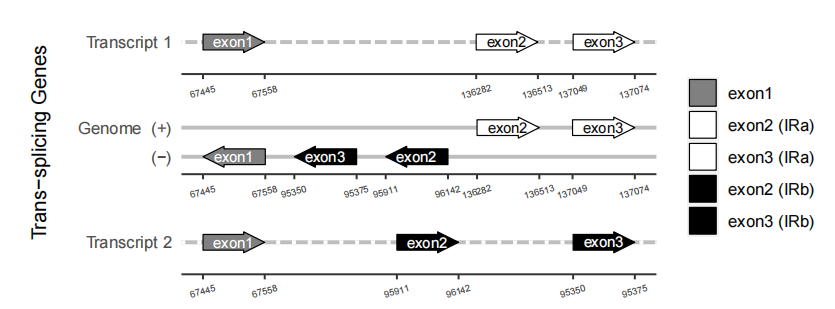

Supplement: GraphicalAbstractS2.png [file TMDN_A_2392762_SM1866.png]

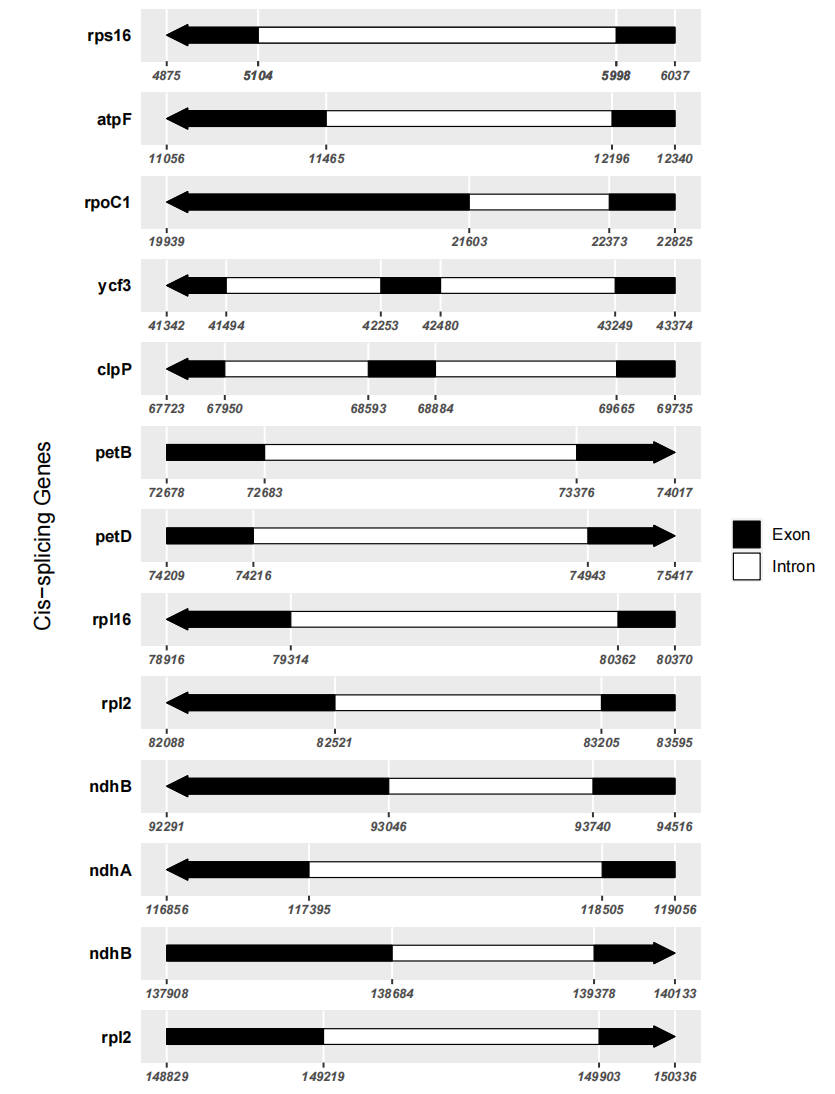

Supplement: GraphicalAbstractS1.png [file TMDN_A_2392762_SM1865.png]

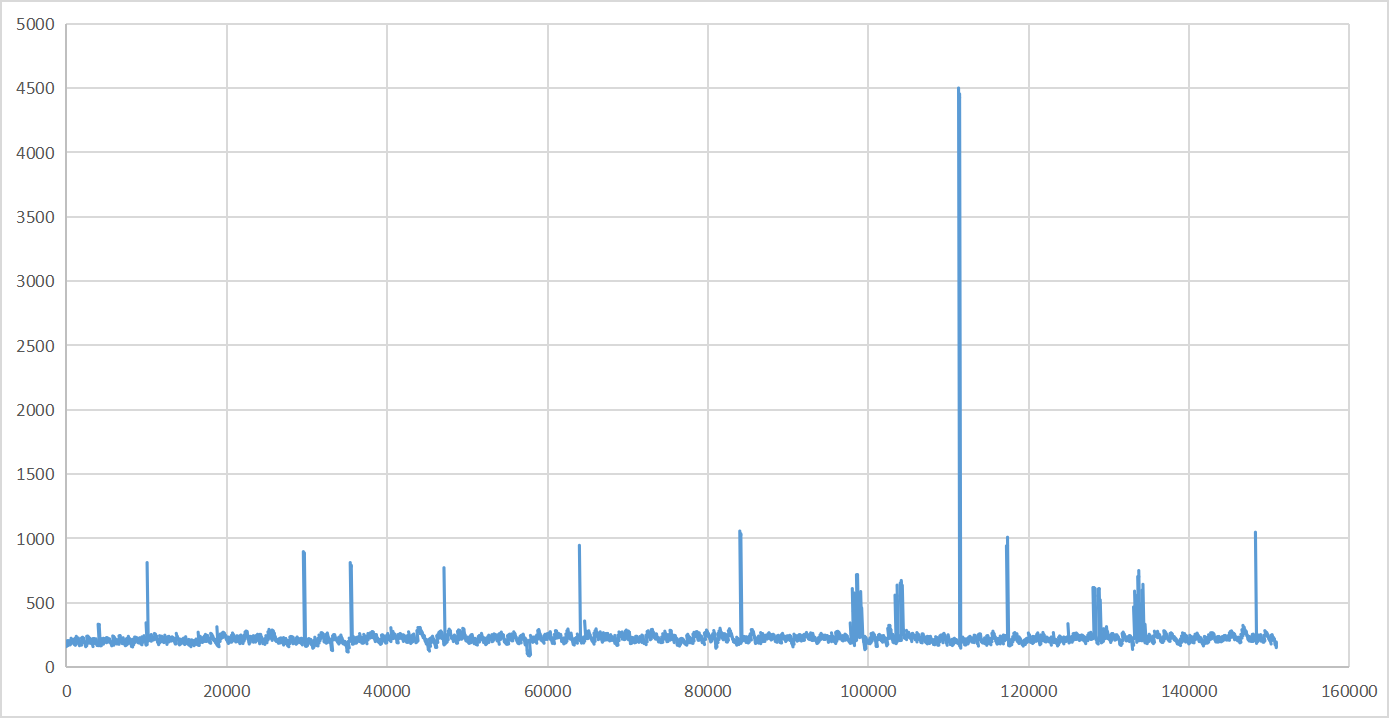

Supplement: GraphicalAbstractS3.png [file TMDN_A_2392762_SM1864.png]
